# Magic Bullets for Mental Disorders: The Emergence of the Concept of an “Antipsychotic” Drug

**DOI:** 10.1080/0964704X.2012.664847

**Published:** 2013-01-16

**Authors:** Joanna Moncrieff

**Affiliations:** Department of Mental Health Sciences, University College London, London, UK

**Keywords:** antipsychotics, history of psychopharmacology, psychiatric therapeutics, treatment specificity

## Abstract

When “antipsychotic” drugs were introduced into psychiatry in the 1950s, they were thought to work by inducing a state of neurological suppression, which reduced behavioral disturbance as well as psychotic symptoms. This view was reflected in the name “neuroleptic.” Within a few years, however, the idea that the drugs were a disease-specific treatment for schizophrenia or psychosis, and that they worked by modifying the underlying pathology of the condition, replaced this earlier view, and they became known as “antipsychotics.” This transformation of views about the drugs’ mode of action occurred with little debate or empirical evaluation in the psychiatric literature and obscured earlier evidence about the nature of these drugs. Drug advertisements in the *British Journal of Psychiatry* reflect the same changes, although the nondisease-specific view persisted for longer. It is suggested that professional interests rather than scientific merit facilitated the rise of the disease-specific view of drug action. The increasing popularity of atypical antipsychotics makes it important to examine the origins of the assumptions on which modern drug treatment is based.

The inscription on the Lasker prize awarded in 1957 read, “for the introduction of chlorpromazine into psychiatry and for the demonstration that a medication can influence the clinical course of the major psychoses” (Deniker, 1989, p. 253). These words encapsulate the importance that was attributed to finding a physical intervention for psychiatric disorders that was more than just a sticking plaster and to finding something that could change the very nature of the disorder itself. Although chlorpromazine and other early antipsychotics were thought to be uniquely useful in the treatment of psychiatric conditions, few people believed at this time, however, that the drugs acted on the disease process itself. The idea that they acted in a disease-specific or “disease-centered” manner developed later, but as the inscription suggests, psychiatry was already aspiring towards such a treatment.

With the discovery of anti-infective agents like Salvarsan and the sulphonamides, and the isolation and synthesis of the hormones insulin and thyroxine, medicine in the twentieth century became strongly associated with the notion that medical treatment could target the underlying disease. “Cure by specific therapy” became “the only really proper sphere for the physician” (Pellegrino, 1979, p. 255). This new orientation, which replaced the humoral model of disease, placed greater power in the hands of the physician. A specialized knowledge of the mechanics of the body and how it could go wrong, which was not available to the layman, was required to identify a particular disease and its corresponding cure or treatment. The greater efficacy of specific treatments justified the greater power and prestige accorded to the medical profession (Rosenberg, 1986) and medicine has continued to deliver drugs that target the physiological processes underlying particular diseases or symptoms.

Although there is much debate about the inclinations of psychiatry in the mid-twentieth century, and whether and to what extent it was dominated by psycho-analytical or biological theories of mental disorder, most commentators agree that, from the 1950s, there was an emphasis on biological theories and treatments (Shorter, 1997; Healy, 2002). One of the major developments in psychiatry at this time was the introduction of a new range of psychopharmaceuticals, starting with chlorpromazine and followed by a number of other drugs, that came eventually to be known as “antipsychotics.” Although various types of sedative drugs had been used prolifically in psychiatry prior to the introduction of chlorpromazine, they were not regarded as worthy of attention and were viewed as acting essentially as chemical restraints (Braslow, 1997; Moncrieff, 1999b). In contrast, chlorpromazine and other drugs introduced subsequently appeared to follow in the footsteps of physical treatments like insulin coma therapy and electroconvulsive therapy (ECT), which had been introduced to great acclaim from the 1930s and which inspired the view that an intervention applied to the body could alter the course of a mental condition, rather than simply containing disturbed behavior (Moncrieff, 1999b).

The new drugs were not at first, however, credited with disease-specific effects. Early publications suggest they were understood as producing a particular drug-induced state, a special sort of sedation, that was useful in situations involving agitation, confusion, disturbance, or psychosis (Moncrieff, 2008b; Whitaker, 2002). Elsewhere, I have referred to this view as the “drug-centered” model of psychiatric drug action to emphasize how psychiatric drugs are psychoactive substances like alcohol and opiates and that modify normal feelings and behavior, producing an altered state of consciousness. According to this model, the altered, drug-induced state is understood to suppress or mask the symptoms of mental distress or disturbance (Moncrieff & Cohen, 2005).

In contrast, current discussions of psychiatric drugs, including antipsychotics, are based on the assumption that they act in a disease-specific or “disease-centered” manner (Moncrieff & Cohen, 2005; Moncrieff, 2008b). According to this view, psychiatric drugs are effective because they reverse or modify the physiological processes that are hypothesized to produce particular mental symptoms in the same way that drugs work in other areas of medicine.^1^ According to this view, the altered state or psychoactive effects that drugs produce are merely incidental “side effects” and irrelevant to their therapeutic actions. Although assumptions about the mechanism of action of drug treatment in psychiatric literature remain largely implicit, current textbooks provide detailed coverage of the effects of drugs on various neurotransmitter systems and of theories of underlying neurotransmitter abnormalities in psychiatric disorders, such as the dopamine hypothesis of schizophrenia, but omit any serious discussion of the psychoactive effects of drugs (the way they modify normal feelings and behavior) and their impact on mental symptoms (see, for example, *The New Oxford Textbook of Psychiatry*; Meltzer & Babo, 2009). The latest edition of the principle American textbook of psychiatry, although suggesting that classifying drugs according to “their major indications is problematic” (Sussman, 2009, p. 2967), stresses that “mental disorders are true medical conditions that can benefit from drug therapy in the same way that diabetes, asthma and hypothyroidism, and other chronic disorders are responsive to medication” (Sussman, 2009, p. 2985).

Literature produced for public consumption sometimes makes more explicit claims. A leaflet on schizophrenia produced by the American Psychiatric Association in 1996 suggested that antipsychotic drugs “help bring biochemical imbalances closer to normal” (American Psychiatric Association, 1996, p. 7) and leaflets on antidepressants make similar claims (Royal College of Psychiatrists, 2007). Information produced by drug companies also stresses the rectification of underlying chemical imbalances as the mechanism of action of antipsychotics and other drugs. Eli Lilly, for example, describes how “antipsychotic medicines are believed to work by balancing the chemicals found naturally in the brain” (Eli Lilly, 2011).

The emergence of the disease-specific view in the 1950s and 1960s has been noted by several authors (Healy, 2002; Whitaker, 2002; Gelman, 1999). In the current article, I present a detailed exploration of the evolution of ideas about the mechanism of action of the new “antipsychotic” drugs between the 1950s and the 1970s. To examine the psychiatric perspective, I analyzed psychiatric papers, textbooks, conference proceedings, and reminiscences of eminent psychopharmacologists, including the three volumes of interviews conducted by David Healy (1996, 1999, 2000). I also examined advertisements published in the *Journal of Mental Science* (later named the *British Journal of Psychiatry*) yearly between 1955 and 1960 and then every five years up to 1980 to ascertain how antipsychotics were presented as working in promotional material. Due to their relative independence from the prevailing academic consensus, advertisements, as well as reflecting commercial concerns, may provide useful clues as to how drugs were used and perceived in everyday practice. Using these sources I describe early ideas about the actions of the new drugs and the development of the view that they exerted disease-specific effects in certain conditions, particularly schizophrenia or the broader category of psychosis. I also contrast the emerging academic and professional consensus on disease-specificity with the more varied and opportunistic stance of the pharmaceutical industry.

## Early Accounts of the New Drugs

Judith Swazey's detailed account of the development of chlorpromazine and its introduction into psychiatry illustrates how early reports provided detailed descriptions of the characteristic mental and physical effects induced by the drug (Swazey, 1974). These descriptions consistently emphasize the emotional indifference produced by the drug or the related idea of a reduction in responsiveness to external events. Laborit, the naval surgeon who used the drug in his anesthetic “cocktail,” describes the “twilight state” induced by the drugs (Laborit & Huguenard, 1951), which he notes later is characterized by a “disinterestedness” in external events (Laborit, Huguenard, & Alluaume, 1952). French psychiatrists Jean Delay and Pierre Deniker, who heard about Laborit's use of chlorpromazine and started using it in their psychiatric patients, described its “psychic effects” (Delay, Deniker, & Harl, 1952, p. 115) in a series of conference presentations, later published as papers in 1952. In their report of the treatment of 38 patients with “excited states,” they described the drug-induced state in some detail, concluding that

The apparent indifference, or delay in response to external stimuli, the emotional and affective neutrality, the decrease in both initiative and preoccupation without alteration of conscious awareness or in intellectual faculties constitute the psychic syndrome due to treatment. (Delay & Deniker, 1952, pp. 503–504)

Other psychiatrists who reported on the use of chlorpromazine in these early days made similar observations. Parisian neurologists, Sigwald and Bouttier, who gave the drug to their neurological outpatients, noted how it reduced the distress associated with delusions and hallucinations and lessened the “imperative character” of obsessional thoughts (Sigwald & Bouttier, 1953, p. 176). British psychiatrist Anton-Stephens described its main effects as “somnolence” and “psychic indifference,” which he deemed to be most useful in “quieting disturbed behaviour” (Anton-Stephens, 1954, p. 557). In their report of the first clinical trial of chlorpromazine, Joel and Charmian Elkes of Birmingham commented on how patients on chlorpromazine became “quieter and more amenable to suggestion by the nursing staff” and, although their psychotic symptoms persisted, they were “less disturbed” by them (Elkes & Elkes, 1954, p. 563). Heinz Lehmann, who introduced chlorpromazine to Canada, noted that the drug made patients lethargic and that “patients under treatment display a lack of spontaneous interest in their environment, yet are easily accessible and respond as a rule immediately and relevantly to questions, even if awakened from sleep” (Lehmann & Hanrahan, 1954, p. 230).

In 1954, two separate researchers, Professor Hans Steck of Lausanne and German psychiatrist Hans Joachim Haase, provided the first unambiguous descriptions of chlorpromazine's effects on the neurological motor system, now generally referred to as extra-pyramidal side effects. They both remarked on the similarity between the drug-induced effects: the decreased movement, reduced facial expression, loss of initiative and muscular rigidity, and the symptoms of Parkinson's disease. They also described the drug-induced agitation known as akathisia (Steck, 1954; Haase, 1954). Within a couple of years, these neurological effects were well recognized, and other neurological effects had been identified including muscular spasms and involuntary movements (Hollister, 1957; Kline, 1956).

## The Concept of a Neuroleptic

In early attempts to define the nature of drugs like chlorpromazine and reserpine, Delay and Deniker stressed two aspects of the drugs’ effects: the peculiar nature of the sedation, which, in contrast to barbiturates, produced a sleep from which people could be easily roused, and the state of disinterest produced by the drugs (Deniker, 1956; Delay & Deniker, 1956). They saw the motor effects as incidental rather than primary, but the sedation and indifference were also regarded as neurological effects by virtue of being induced by drug action on the brain and nervous system. In early papers, therefore, they referred to chlorpromazine as a “neuroplegic” drug (from the Greek to paralyze),^2^ and, in 1955, they replaced this term by the term “neuroleptic” (from the Greek to seize), still emphasizing the sedative and emotional effects of the drug, rather than its motor effects (Delay & Deniker, 1955).

In 1957, Delay and Deniker came across a new drug called prochlorperazine, which was being tested in psychiatric patients by Pierre Brousolle and others working in Lyon. Prochlorperazine was a phenothiazine like chlorpromazine but was much less sedating in its actions and produced dramatic neurological reactions, described at the time as “excito-motor” effects, including muscular spasms, severe akathisia, and other movement abnormalities both in psychiatric patients, and in military personnel who were given the drug against sea sickness (Delay, Deniker, Green, & Mordret, 1957; Comite Lyonnais de Recherches Therapeutiques en Psychiatrie, 2000). Deniker later recalled that the experience of using prochlorperazine persuaded him and Delay that characteristic abnormal motor effects were an intrinsic part of the neurological state produced by the drugs (Deniker, 1989).

Deniker outlined the theory behind the idea of a neuroleptic most explicitly in a paper published in 1960, entitled “Experimental Neurological Syndromes and the New Drug Therapies in Psychiatry” (Deniker, 1960). In this paper, he suggested that the neuroleptics act through inducing a neurological syndrome, somewhat similar to the postencephalitis type of Parkinson's disease. As in his other publications, he described in some detail the way the drug made patients look and behave. Thus, he noted how patients “look as if they have been turned to stone, they are usually indifferent to themselves and their environment, they are stuporous or prostrate, even before the clinical symptom of hypertonia (rigidity) appears” (Deniker, 1960, p. 96). Although Deniker admitted that he had previously found chlorpromazine useful in doses low enough not to cause obvious physical symptoms, in this paper, he asserted the notion that it was necessary to “resolutely and systematically aim to produce neurological syndromes to get better results than can be obtained when neuroleptic drugs are given at less effective doses” (Deniker, 1960, p. 100).

German psychiatrist, Hans-Joachim Haase also proposed that the therapeutic effects of the new drugs consisted of a mild version of the Parkinson disease-like syndrome they induced (Haase, 1956). Later, he coined the term “neuroleptic threshold” to indicate the dose of a drug that could achieve therapeutically useful, mild neurological suppression, without producing frank Parkinsonian symptoms (Haase & Janssen, 1965).

Similar views were proposed by American psychiatrist, F. A. Freyhan, speaking at a symposium held in Switzerland in 1957 (Freyhan, 1959). He stressed the belief that the effects of the new drugs were not specific to any diagnostic group but acted on signs of overarousal, excitement, and abnormal preoccupations due to their ability to reduce movement and initiative and blunt emotions. Like Deniker and Haase, Freyhan suggested that the drugs useful or therapeutic effects were on a continuum with their obvious extra-pyramidal or Parkinsonian effects:

From the beginning it was evident that no lines of demarcation could be drawn between therapeutic degrees of reduced psychomotor activity and early symptoms of parkinsonism…. What we witnessed were gradual transition from hypermotility to hypomotility, which, in a certain proportion of patients, progressed to the more pronounced degrees of parkinsonian rigidity. Clinical evidence therefore, indicated that the therapeutic function of chlorpromazine and reserpine could not be separated from their modifying influence on the function of the subcortical motor system in transacting volitional, affective and intentional functions. (Freyhan, 1959, p. 10)

Psychiatrists in England and Canada echoed these views (Denham, 1965; Sarwer-Foner, 1960), and there was also support for the view that inducing the neurological symptoms of Parkinsonism was necessary to achieve the therapeutic benefits of the neuroleptics (Denham & Carrick, 1960; Karn & Kasper, 1959; Flugel, 1959). The “drug-centered” viewpoint was summarized by participants at a symposium held in 1955, who concluded that chlorpromazine could be used to “attain a neuropharmacologic effect, not to ‘cure’ a disease” (Smith Kline & French Laboratories, 1955, p. 158 as cited in Whitaker, 2002, p. 146).

## From Tranquilizers to Antipsychotics

The idea of a neuroleptic was never accepted to the same degree in America. There, drugs like chlorpromazine and reserpine were referred to as “tranquilizers,” and later as “major tranquilizers,” to distinguish them from meprobamate, and the newly launched benzodiazepine, Librium, which were increasingly labelled as “minor tranquilisers.”^3^

As early as 1954, participants at a psychiatric symposium in Washington, DC, clearly stated that they believed chlorpromazine and reserpine were attacking the “underlying schizophrenic process” (Kinross-Wright, 1956) and exerting a “specific effect on the basic schizophrenic mechanisms” (Sainz, 1956). A participant, who did not share this view, bemoaned the tendency to jump from the effects of drugs to making generalizations about the genesis of psychotic behavior (Meyers, 1956). In 1955, the President of the Society of Biological Psychiatry of the United States reflected that the new drugs were of a “different order” from previous drugs, and that they could “wipe out the symptoms of psychotic patients just as internists can use insulin for the elimination of the symptoms of diabetes” (Himwich, 1955, p. 421).

At the same symposium in 1957 in which Freyhan outlined his drug-centered view of the action of the new tranquilizers, Heinz Lehman set out the first explicit, although brief, disease-centered view of the actions of chlorpromazine and similar drugs (Lehmann, 1959). As well as attempting to classify the new drugs by their physiological effects on the nervous system, in other words, whether they exerted “inhibiting,” “exciting,” or psychotomimetic effects, he suggested that the effects of drugs could be divided into those that were “curative,” “corrective,” and “symptomatic.” Whereas, a curative substance was one that reversed the original cause of the disease, like antibiotics, a corrective was one that attacked a “nucleus of symptoms that is fairly close to the primary disturbance,” although the primary cause of the condition need not be known. Examples of correctives in medicine were insulin for diabetes and digitalis for heart disease, and Lehmann identified chlorpromazine and reserpine as “typical corrective agents in a number of acute and chronic psychotic conditions” (1959, p. 22). He contrasted their effects to symptomatic treatments, like morphine for pain, barbiturates for insomnia, and chlorpromazine when it was used for the control of behavioral excitement, which only affected symptoms that were “rather remote and indirect manifestations” of the disease process (Lehmann, 1959, p. 23).

Heinz Lehmann has said that he introduced the term “antipsychotic” at a Canadian Medical Association meeting in 1956, but that he meant it more metaphorically than literally at this time (Lehmann, 1993). The first paper listed on Medline to use the term was published in 1962,^4^ in which two psychiatrists distinguished an “antipsychotic drug” that “antagonises major psychotic symptoms” from other tranquilizers, which ameliorate the “symptom of anxiety” (Mapp & Nodine, 1962, p. 458). Despite this distinction, however, they did not attribute “curative” or “corrective” properties (in Lehmann's terms) to the drugs they were describing but expressed confidence that “more specific agents” would be discovered in the future that would act on the “etiology of the symptoms rather than the symptoms themselves” (Mapp & Nodine, 1962, p. 463).

One of the most influential figures to promote the disease-centered theory of drug action was American psychiatrist Nathan Kline (Healy, 2002). In 1959, he described chlorpromazine and reserpine as “ataraxics,” meaning, according to Kline, “freeing from turmoil and confusion” (Kline, 1959, p. 398). He also stated that the really unique property of the drugs was to “remove, reverse, restrict or inhibit” mental symptoms, including those of psychosis (Kline, 1959, p. 398). But it was his characterization of what later came to be known as “antidepressants” that expressed disease-specific ideas most explicitly. Kline distinguished these drugs from stimulants, which were known to cause physical arousal and to elevate mood to the point of euphoria. In contrast, he said, drugs that he called “psychic energizers” (later referred to as antidepressants), which had previously been described as stimulant-type substances (Crane, 1956), did not cause euphoria but normalized mood in those who were depressed (Kline, 1959).

In 1964, a well-known study funded by the United States National Institute for Mental Health (NIMH) claimed to have demonstrated the disease-specific effects of the new drugs for the treatment of schizophrenia (National Institute of Mental Health Psychopharmacology Service Center Collaborative Study Group, 1964). The trial, which involved chlorpromazine and two other recently introduced drugs, found that all three drugs improved a range of symptoms greater than the placebo. Since these symptoms included not just excitement, agitation, and anxiety but what were regarded as more fundamental schizophrenic symptoms including incoherence of speech, social withdrawal, and apathy, as well as auditory hallucinations and persecutory delusions, the authors concluded that “the phenothiazines should be considered to be ‘anti-schizophrenic’ in the broad sense. In fact, it is questionable whether the term ‘tranquiliser’ should be retained” (National Institute of Mental Health Psychopharmacology Service Center Collaborative Study Group, 1964, p. 257). They also noted that the drugs tested varied in their proclivity to produce extrapyramidal or Parkinsonian effects, and yet, because all were equally effective, they suggested that “the therapeutic properties of these drugs may be quite independent of their tendency to produce side effects” (National Institute of Mental Health Psychopharmacology Service Center Collaborative Study Group, 1964, p. 255).

Sheldon Gelman has described how the report of this trial encapsulated a new psychiatric “vision” that suggested that drug treatment was effective and specific with relatively trivial adverse effects (Gelman, 1999). The trial was not designed to demonstrate how the drugs worked, however. In particular, there was no comparison with other sedatives, no consideration of the nature of the altered state produced by the drugs and its impact on symptoms, and the neurological “extrapyramidal” effects were already conceptually distinguished from the drugs’ therapeutic effects and were referred to as “side effects.” The concept of the “neuroleptic threshold,” which suggests that drugs with different propensities to cause extrapyramidal effects can exert therapeutic neurological effects at variable, “threshold” doses, was ignored.

## The Ascendancy of the Disease-Centered View

The idea that drugs like chlorpromazine could suppress a range of psychotic symptoms does not in itself necessarily imply a disease-centered view of drug action. But the language used, the separation and minimization of neurological “side effects,” and the broad endorsement of the drugs suggest that they were in the process of being transformed into drugs that were comparable to other, specific medical treatments.

This transformation happened with almost no direct discussion or debate. Although a few figures like Deniker and Haase continued to reiterate a drug-centered view, in general, what is striking is that this older conception of drug action simply faded away. Descriptions of the neurological and general effects induced by neuroleptics and other psychiatric drugs, such as antidepressants, disappeared from the literature (Moncrieff, 2008a) and were henceforth referred to, if at all, only as “side effects,” which were regarded as an incidental nuisance, rather than an intrinsic part of a drug's action. Almost no papers discussed the relative merits of different theories of drug action or attempted to justify the disease-centered model, and no research was set up to evaluate different models. When textbooks started to present the disease-centered view, there was no acknowledgement that there were alternative explanations of how antipsychotics might work.

Psychiatric textbooks tentatively started to convey a disease-centered view of the nature of antipsychotic drugs from the 1960s. The principle British textbook of the period was edited by two well-known socially inclined psychiatrists. In the 1962 edition, they hesitated to call the new tranquilizers “specific” but felt that “they appear to do more than tranquilise” (Henderson & Gillespie, 1962, p. 350). A more biologically oriented textbook was bolder, suggesting that the “drugs penetrate much closer to the site of mechanism of the disease itself than any other procedure applied hitherto” (Mayer-Gross, Slater, & Roth, 1960, p. 386). By 1969, the section on drug treatment of schizophrenia concluded that “phenothiazine drugs cannot be regarded as being merely symptomatic therapies in the same sense as analgaesic drugs” (Mayer-Gross, Slater, & Roth, 1969, p. 330).

The *Companion to Psychiatric Studies* was published for the first time in 1973 and became one of the most respected British textbook for the next few decades. The general chapter on pharmacology in the first edition presented a drug-centered account of the effects of neuroleptics, suggesting that the drugs damp down responses to stimuli and reduce spontaneity. Following Delay and Deniker, it described the drugs’ unique quality as their ability to induce sedation without sleep (Roberts, 1973). The chapter on the treatment of schizophrenia, however, written by a different author, assumed that the drugs act in a disease-centered manner (Smythies, 1973). The chapter refers to the drugs as being “anti-schizophrenic” and it is asserted that “there is now abundant evidence from well-controlled trials that the phenothiazines and butyrophenones exert a specific therapeutic effect in schizophrenia, and that the term ‘tranquiliser’ is a misnomer” (Smythies, 1973, pp. 281–282). The author also speculated on the role of dopamine and serotonin in the drug's mechanism of action and suggests that if this could be clarified, then the “biochemical lesion of schizophrenia” (Smythies, 1973, p. 282) would be elucidated.

The largest American textbook of the mid-twentieth century, *The American Handbook of Psychiatry*, first published in 1959 and again in 1966, made no explicit comments about the mode of action of the drugs, although effects were divided into therapeutic effects and side effects, and there was no description of the altered state the drugs induce and how this might impact on symptoms (Hoch, 1959; Malitz & Hoch, 1966). In the second edition, published in 1975, it was claimed that “specific pharmacological treatments for the major psycho-pathological states have become available” and that these agents were able to elucidate the biochemical origins of these disorders (Maas & Garver, 1975, p. 427). In the chapter called “Antipsychotic Drugs,” written by Jonathan Cole, the lead researcher on the NIMH study, and John Davis another well-known academic, it was asserted that the drugs are “most correctly called ‘antischizophrenic’ or ‘antipsychotic’ drugs. They do not, in any real sense, produce a state of tranquillity in either normal or psychotic individuals” (Davis & Cole, 1975, p. 446). Research on dopamine was described, and it was speculated that the drugs may exert their effects by reducing dopamine activity.

By 1980, the first edition of what has subsequently become the major American textbook of psychiatry unequivocally asserted the disease-centered position. The section on antipsychotic drugs, written by John Davis, stated “antipsychotic drugs have a normalizing effect. They lessen the typical schizophrenic symptoms, such as hallucinations and delusions. They also normalize various other abnormal behaviors” (Davis, 1980, p. 2260). Davis also stressed that “there is a clear cut difference between their sedative and antipsychotic properties” and, in contrast to earlier views, suggested “it is a mistake to think of those drugs as a special type of sedative” (Davis, 1980, p. 2260). Solomon Snyder, author of the general section on psychopharmacology, asserted that the drugs “exert a selective anti-schizophrenia action,” following with a detailed description of their actions on the dopamine system (Snyder, 1980, p. 161).

None of the textbook sections provide any citations or evidence to support the disease-centered view of drug action, although many are reminiscent of points made in the NIMH study report (National Institute of Mental Health Psychopharmacology Service Center Collaborative Study Group, 1964).

A rare discussion of alternative understandings of drug action by Nathan Kline in 1969 reveals how much was felt to be at stake by acknowledging the drug-centered point of view. In his 1959 paper, Kline rejected the concept of a neuroleptic, with the reasoned justification that the mode of action the term suggested was unproven. Ten years later, he dismissed a World Health Organization report on psychopharmacology that had accepted the concept, presumably under Deniker's influence, with invective and bluster. He described the report as being “deGaulling” in its “capitulation” to the French point of view, and as using a “weasling excuse” to defend its position (Kline, 1969). Although characteristic of Kline's forthright personality (Healy, 2002), the language and tone of his comments, far removed from the usual academic form of expression, indicate how challenging the notion of the neuroleptic was by then felt to be, and how strongly Kline wanted to bury it.

## The Portrayal of the New Drugs in Advertisements

Although advertisements in the *British Journal of Psychiatry* also illustrate the transformation of views about the nature of the new drugs and their mechanism of action, the desire to appeal to a large market meant that some substances continued to be marketed for their sedative actions in a range of conditions or situations, well into the 1970s.

The first advertisement for a drug that would now be considered an antipsychotic was published in 1956 and concerned reserpine. It described reserpine in clearly drug-centered terms as having “remarkable calming and relaxing action … capable of wide application in mental illness” (Reserpine advertisement, 1956). In 1960, a four-page color advertisement for Melleril, featuring a large picture of a calm and beautiful lake, also stressed the “psychosedative” action of the drug, describing it as “the tranquiliser pure and simple” (Melleril advertisement, 1960). All the “antipsychotic” drugs featured in the 1960 editions of the journal, with the exception of Stelazine (trifluoperazine), which was only advertised for use in schizophrenia, were recommended for a broad spectrum of psychiatric conditions, including anxiety, agitation in the elderly, and childhood behavior problems, as well as the treatment of schizophrenia.

By 1965, hints of a disease-centered view appeared, with the use of terms like “psychocorrective” (Largactil or chlorpormazine; Largactil advertisement, 1965) and “profound” (Serenace or haloperidol; Serenace advertisement, 1965) to describe the action of the drugs in the management of schizophrenia. One advertisement for Stelazine, which was first printed in February picturing a troubled-looking elderly man, with the caption “withdrawal” (Stelazine advertisement, 1965a), by August had added a second picture of a younger man, looking comfortable and relaxed and lying on a couch reading a book. The clear implication was that Stelazine restores normality (Stelazine advertisement, 1965b). Most of the drugs, however, were still aimed at a broad range of situations and there were still references to drug-induced effects such as calming, control, and sedation (Melleril advertisement, 1965).

In 1970, Neulactil (pericyazine) was being recommended for “behaviour disorders at all ages” (Neulactil advertisement, 1970), but advertisements for Triperidol (trifluperidol; Triperidol advertisement, 1970), Serenace (Serenace advertisement, 1970), and Melleril were specifically aimed at schizophrenia (there were no adverts for Stelazine in this year). The Melleril advertisement, which consisted of two color pages, claimed that Melleril “strikes promptly at the target symptoms” and referred to the drug as an “anti-psychotic.” The effect on target symptoms, which was said to occur after seven days, was also distinguished from the immediate sedative or “tranquilizing effect” of the drug (Melleril advertisement, 1970).

In 1975, Fentazin (perphenazine; Fentazin advertisement, 1975) and Serenace (Serenace advertisement, 1975) were still being recommended in a variety of situations, but most advertisements concerned long-acting, injectable depot medications that were aimed exclusively at people with schizophrenia or psychotic disorders. None of the advertisements mentioned any psychoactive properties of the drugs. In June 1975, a new drug called Redeptin was advertised for the first time, with explicit claims of a disease-centered action. It was said to have a “specific action on dopamine receptors” (see below) and was referred to as having an “antipsychotic” action (Redeptin advertisement, 1975).

By 1980, only the newly launched Droleptan (droperidol), which was still described as a “major tranquilizer,” was being advertised for nonspecific purposes: the “rapid control of acute agitation” (Droleptan advertisement, 1980). A large number of advertisements for other antipsychotic agents were aimed specifically at the treatment of schizophrenia, but none provided any explanation of the mechanism of action of the drugs in any terms.

## The Dopamine Hypothesis of Schizophrenia

The dopamine hypothesis of schizophrenia is clearly important to the current discussion, although a detailed history of the hypothesis would constitute another article in its own right. The origins of the dopamine theory of schizophrenia are often dated to a paper published in 1963 by the renowned Swedish pharmacologist Arvid Carlsson. In this paper, he showed that haloperidol and chlorpromazine affected the synthesis of catechol amines, including dopamine (Carlsson & Lindqvist, 1963). In 1966, a Dutch researcher, van Rossum, showed that haloperidol, an antipsychotic developed by Janssen in 1958, reduced dopamine activity by blocking the dopamine receptor and tentatively suggested that this finding may have implications for the etiology of schizophrenia (Van Rossum, 1966). Although the dopamine hypothesis was not clearly articulated until the 1970s it appeared to be widely known before this time (Moncrieff, 2009). Its origins lay in the relatively specific effects—in pharmacological terms—of haloperidol on dopamine receptors, and effects that were not as clear with the broader acting drugs like chlorpromazine and reserpine. By inferring a theory of the pathology of schizophrenia from the action of the drugs used to treat it, the dopamine hypothesis *assumes* that the drugs act in a disease-centered way. References to the theory in textbooks and advertisements, however, indicate it was regarded as supporting the disease-centered theory of drug action. Regardless of whether or not there was any empirical evidence for the idea, the speculation that the dopamine blocking effects of antipsychotics indicated the origins of the condition of schizophrenia strengthened the emerging idea that the drugs acted by reversing an underlying biochemical abnormality.

## Discussion

The psychiatrists who started using the new drugs in the 1950s welcomed them enthusiastically, regardless of whether they understood them according to the drug-centered model, as novel psychotropic substances with a uniquely useful profile of action, or whether they believed them to be a new, and possibly the first, disease-specific treatment for serious mental illness (Moncrieff, 1999a). Delay and Deniker were as convinced of the importance of the discovery of the new drugs as anyone else in psychiatry (Delay & Deniker, 1955).

Interest in the new drugs continued but, in the space of a few years, the way they were understood changed fundamentally. From being viewed as drugs that induced a characteristic and useful state of neurological suppression, they were transformed into a disease-specific treatment for schizophrenia. The altered mental and physical state induced by the drugs came to be seen as irrelevant to their therapeutic action. The more subtle emotional and behavioral effects disappeared from the literature altogether. The characteristic “extrapyramidal” motor effects, although recognized as a marker for drugs with antipsychotic action, came to be seen as quite distinct from the desired effects and were relegated to the status of “side effects.” This transformation was not inspired by the results of empirical research, or even informed debate, however. The idea that the drugs were disease specific simply appeared to replace the older view, which subsequently ceased to be even acknowledged, suggesting that the process of transformation was driven by factors other than the scientific merits of the disease-centered view.

The drivers of this metamorphosis included the psychiatric profession, which, since it identified itself as a profession in the nineteenth century had felt the continued need to justify the role of medicine in caring for people deemed at the time to be “insane” (Rogers & Pilgrim, 2001; Scull, 1993). This insecurity continued into the twentieth century and it was in this context that physical treatments like ECT and lobotomy were welcomed into psychiatry (Moncrieff & Crawford, 2001). The new drugs helped bring psychiatry further into line with existing medical practice, and contemporaries were delighted that the drugs made “the mental hospital a medical institution in the minds of the public” (Overholser, 1956). In 1975, Jonathan Cole commented that “for the first time, public mental institutions could be regarded as true treatment centres, rather than as primarily custodial facilities” (Davis & Cole, 1975, p. 442). Deniker, too, described approvingly how the new drugs had strengthened the medical and scientific approach in psychiatry (Deniker, 1970).

The drug-centered view of how psychiatric drugs might work was not, however, a model that could be easily married with the increasingly specific nature of other medical treatments. Moreover, the suggestion that the drugs acted by neurological suppression or restriction provided ammunition for critics like Thomas Szasz, who portrayed the drugs as chemical strait jackets that were used in the project of psychiatric social control (Szasz, 1957). Szasz published *Myth of Mental Illness* in 1960, and psychiatry came under attack from various sources through the 1960s and 1970s as the antipsychiatry movement progressed. Budgetary constraints and the introduction of managed care in the United States also increased competition from nonmedical therapists (Wilson, 1993). The profession reacted by attempting to strengthen its medical and scientific credentials, and the idea of the specificity of drug treatment became a central part of that endeavor (Moncrieff, 2008b). The specificity of drug treatment was cited in response to the Rosenhan experiment, for example (Spitzer, 1976), in which normal volunteers obtained admission to mental hospitals and were diagnosed with schizophrenia, apparently calling into question the validity of psychiatric diagnosis (Rosenhan, 1973). More importantly, however, large areas of biochemical and pharmaceutical research were instigated that in itself, regardless of its results, helped portray psychiatry as a credible and thriving scientific enterprise.

Other factors are likely to have influenced the rise of the disease-centered view of drug treatment, however. The rise of a recreational drug culture has been suggested to have increased the inclination to distinguish between “drugs” and medicines and to play down the ability of medical drugs to produce altered mental states (DeGrandpre, 2006). Regulatory changes passed during the 1960s are also likely to have been influential, particularly the Kefauver-Harris amendment to the Food and Drug Act in the United States that passed in response to the thalidomide tragedy (Anderson, 2005). This amendment mandated companies to provide evidence of efficacy as well as safety in order to obtain a license for their products. The need to specify each situation in which a drug was thought to work reduced the ability of companies to market drugs across a range of indications and encouraged association between particular drugs and particular disorders.

Regulatory restrictions, as well as professional directions, are likely to have influenced the changing portrayal of antipsychotic drugs in advertising. Up to the 1970s, advertisements for many substances reflected the marketing advantages of a drug-centered model in aiming at a broad range of psychiatric situations. By the end of the 1970s, however, the industry appeared to have come into line with the professional view, and the drugs were mostly portrayed as specific treatments for schizophrenia with references to their drug-induced effects expunged.

In the present day, by contrast, it is the pharmaceutical industry that most explicitly promotes the disease-centered view of antipsychotics and other psychiatric drugs with material on company Web sites frequently referring to the idea that drugs reverse chemical imbalances (Moncrieff, 2006). The promotion of this view appears to contradict the growing tendency for antipsychotics to be used in a variety of disorders including depression and in situations like insomnia and agitation in the elderly where drug-induced sedation is the intended effect (Verdoux, Tournier, & Begaud, 2010). These apparently conflicting tendencies may be complementary, however. The text on the Eli Lilly Web site cited earlier with its emphasis on how drugs “balance chemicals found naturally in the brain” illustrates how the disease-centered notion that drug treatment is restorative can present a benign view of drug treatment (Eli Lilly, 2011). This message provides a useful counterbalance to unfolding evidence about the adverse effects of these drugs (Ray et al., 2009; Newcomer & Haupt, 2006), which is necessary for the successful promotion of these drugs to people with less severe problems.

The fact that the term “antipsychotic” has become the most popular way of describing the group of drugs I have described indicates the current hegemony of the disease-centered view of their action. With rapidly increasing rates of prescription of antipsychotics, in general, and newer “atypical” antipsychotics, in particular (Ilyas & Moncrieff, 2012), it is timely to remind ourselves that these drugs were not always viewed in this way. The drug-centered view of antipsychotics highlighted the artificial state induced by the drugs and the unwanted knowledge, and potentially dangerous aspects of this state, which has been occluded by the view that they work by reversing disease processes. The fact that our current view of drug action was not established on scientific merit but represents the interplay of professional, commercial, and political concerns suggests the need to reevaluate the basis on which these drugs are used.

## Notes

1 The vast majority of medical drugs target the underlying mechanisms that produce symptoms or disorders. Anti-asthma drugs, for example, work by dilating constricted airways, anticancer drugs by targeting mechanisms of cell proliferation, and pain killers act on the physiological pathways that produce pain.
2 The term “neuroplegic” reflects the influence of Laborit's search for what he called “ganglioplegic” drugs, which were drugs used to paralyze or suppress the ganglia or nervous system (Healy, 2002; Swazey, 1974).
3 The first use of the term “tranquiliser” (or “tranquilizer”) on Medline is in reference to meprobamate in a paper published in March 1956. It was first used to refer to reserpine later the same year (June 1956). The term “major tranquilizer” first appears on Medline in a paper from 1960.
4 Medline was searched from 1955 to 1970 for papers using the exact term “antipsychotic.”
